# Multimodal imaging and machine learning for diagnosis of Parkinson’s disease with cognitive impairment: ASL and QSM as potential biomarkers

**DOI:** 10.3389/fnagi.2026.1752040

**Published:** 2026-01-22

**Authors:** Weimin Qi, Jiang Cheng, Xiuping Zhan, Ting Xu, Shue Gu, Qin Shi, Haining Li

**Affiliations:** 1 Department of Neurology, General Hospital of Ningxia Medical University, Yinchuan, China; 2 Department of Ophthalmology, General Hospital of Ningxia Medical University, Yinchuan, China

**Keywords:** arterial spin labeling (ASL), cognitive impairment, machine learning, magnetic resonance imaging (MRI), Parkinson’s disease, quantitative susceptibility mapping (QSM)

## Abstract

**Objectives:** This study aimed to investigate differences in brain imaging characteristics among patients with Parkinson’s disease with cognitive impairment (PDCI), Parkinson’s disease without cognitive impairment (PDNCI), and healthy controls (HC), and to develop machine learning–based models for the early diagnosis of PDCI. A total of 48 patients with PDCI, 50 patients with PDNCI, and 47 age- and sex-matched healthy controls were enrolled, all of whom underwent magnetic resonance imaging using a 3.0 T MRI scanner. Arterial spin labeling (ASL) was applied to quantify cerebral blood flow (CBF), and quantitative susceptibility mapping (QSM) was used to assess magnetic susceptibility, while cognitive function was evaluated using standardized neuropsychological scales. Group differences were examined using one-way analysis of variance (ANOVA), and seven machine learning classifiers, including random forest (RF), K-nearest neighbors (KNN), and Extreme Gradient Boosting (XGB), were constructed to discriminate among the PDCI, PDNCI, and HC groups. The ANOVA results revealed significant differences in both CBF and magnetic susceptibility between the HC group and the two PD groups, whereas no significant differences were observed between the PDCI and PDNCI groups. Compared with normative data, patients with PDCI exhibited cognitive impairments exceeding 2 standard deviations in the domains of language, attention, and working memory, as well as impairments exceeding 1 standard deviation in visuospatial function, memory, and executive function. Among the machine learning models, RF, KNN, and XGB achieved perfect classification performance, with all evaluation metrics reaching 1.000, indicating excellent discriminative capability. Feature importance analysis identified increased CBF and magnetic susceptibility in regions such as the left precuneus (Precuneus_L) and left postcentral gyrus (Postcentral_L) as key imaging features distinguishing PDCI, and correlation analyses further demonstrated significant associations between cognitive deficits and alterations in CBF and magnetic susceptibility. These findings suggest that ASL- and QSM-derived imaging features have substantial potential as non-invasive biomarkers for the early diagnosis of PDCI, that patients with PDCI exhibit widespread impairments across multiple cognitive domains—particularly in language, attention, and working memory—and that machine learning models integrating multimodal imaging features provide a reliable and effective approach for early diagnosis and may facilitate personalized treatment strategies in Parkinson’s disease, although future studies with larger sample sizes and independent validation cohorts are warranted to enhance the robustness and generalizability of these models.

## 1 Introduction

Parkinson’s disease (PD) is a prevalent neurodegenerative disorder characterized by cardinal motor symptoms, including tremor, rigidity, and bradykinesia. As the disease progresses, an increasing proportion of patients develop cognitive impairment, which may eventually evolve into Parkinson’s disease with cognitive impairment (PDCI). PDCI markedly compromises patients’ quality of life and functional independence and is generally closely associated with disease progression (Goldman and Sieg, 2020). Consequently, elucidating the mechanisms underlying PDCI and improving its early diagnosis are of critical importance for clinical management and prognosis (Aarsland and Kurz, 2010). The clinical presentation of PDCI is heterogeneous and involves multiple cognitive domains, including language, attention, working memory, executive function, and visuospatial abilities (Verbaan et al., 2007). Emerging evidence indicates that PDCI is not merely a result of worsening motor dysfunction but is more likely related to diverse neuropathological alterations, such as cerebral hypoperfusion and abnormal iron accumulation (Qi et al., 2025). Therefore, the identification and quantitative assessment of these pathological changes are essential for the early diagnosis and therapeutic intervention of PDCI.

With advances in neuroimaging technologies, arterial spin labeling (ASL) and quantitative susceptibility mapping (QSM) have emerged as important tools in neuroscience research. ASL is a non-invasive magnetic resonance imaging (MRI) technique that quantifies cerebral blood flow (CBF) by magnetically labeling inflowing arterial blood water, thereby providing critical information on cerebral perfusion (Taso and Alsop, 2024). In patients with PD, ASL has been shown to detect reductions in CBF, particularly in those with cognitive impairment, in whom cerebral hypoperfusion may be closely associated with cognitive decline (Arslan et al., 2020). QSM, by contrast, enables quantitative assessment of the magnetic susceptibility of brain tissue, reflecting pathological alterations such as iron deposition, calcification, and demyelination. In PD patients, especially those with cognitive impairment, abnormal iron accumulation in the brain is considered a key pathological factor contributing to cognitive deterioration (Uchida et al., 2020). Quantitative evaluation of iron deposition using QSM not only provides novel imaging biomarkers for the early detection of cognitive impairment but also facilitates a deeper understanding of the pathological mechanisms underlying PDCI.

In addition, with the rapid advancement of big data analytics and artificial intelligence, machine learning approaches based on imaging features have been increasingly applied in medical diagnosis. Machine learning algorithms, particularly random forest (RF), support vector machine (SVM), and Extreme Gradient Boosting (XGB), have been widely used for medical image classification and disease diagnosis (Prinzi et al., 2024). These approaches can leverage features extracted from ASL and QSM images to construct accurate classification models, thereby providing effective tools for the early identification of PDCI (Shibata et al., 2022). Furthermore, by integrating multimodal MRI features, machine learning models can enhance diagnostic accuracy and enable more precise and personalized assessments of disease status.

Although ASL and QSM have demonstrated considerable potential in PD research, systematic investigations comparing these imaging biomarkers between PD patients with and without cognitive impairment remain limited. Most existing studies have primarily focused on single-modality imaging, whereas research integrating multimodal imaging features is still scarce. Therefore, the present study aims to investigate differences in ASL- and QSM-derived imaging features between PDCI and PDNCI, and to develop machine learning models based on these features to evaluate their clinical value in the diagnosis of PDCI. Clinical and imaging data will be collected from three groups—PDCI, PDNCI, and healthy controls (HC)—to analyze the associations between ASL and QSM features and cognitive function, as well as to compare the diagnostic performance of different machine learning classifiers.

## 2 Materials and methods

This study was approved by the Ethics Committee of the General Hospital of Ningxia Medical University, and written informed consent was obtained from all participants prior to enrollment. The study population consisted of patients with PD who met the inclusion criteria and voluntarily participating HC, who were consecutively recruited from the neurology outpatient and inpatient departments of our hospital between June 2021 and August 2025. PD patients were diagnosed with idiopathic Parkinson’s disease according to the clinical diagnostic criteria of the UK Parkinson’s Disease Society Brain Bank (Postuma et al., 2015) by an experienced neurologist specializing in movement disorders. Participants were excluded if they had a history of cerebrovascular disease, epilepsy, moderate to severe depression, or other neuropsychiatric disorders. All participants were required to be able to tolerate MRI examinations, with no contraindications such as claustrophobia or implanted cardiovascular or cerebrovascular stents, and to have provided written informed consent. Routine brain MRI scans showed no evidence of cerebrovascular lesions, severe demyelination, or other significant structural abnormalities. Participants with a Montreal cognitive assessment (MoCA) score < 26 were classified into the PDCI group (Nasreddine et al., 2005). Ultimately, 48 patients with PDCI, 50 patients with PDNCI, and 47 age- and sex-matched healthy controls were included in the final analysis. Relevant clinical characteristics and comprehensive cognitive assessment data were collected for all participants, as summarized in Table 1.

**TABLE 1:** General information for the PD-CI group, PD-NCI and HC group.

Variable	PD-CI group	PD-NCI group	HC group
Age	60.48 ± 7.30	58.66 ± 8.22	56.62 ± 6.61
Gender (woman/man)	23/25	26/24	25/22
Course of disease	4.56 ± 2.58	3.68 ± 2.80	–
Level of education (years)	7.52 ± 3.53	8.74 ± 3.89	9.60 ± 4.79
UPDRSI	4.21 ± 2.44	3.08 ± 2.23	–
UPDRSII	10.23 ± 4.77	8.76 ± 5.51	–
UPDRSIII	19.98 ± 12.16	17.08 ± 12.39	–
H-Y Median of median (25%, 75%)	2 (1.50, 2.00)	1.5 (1.00, 2.00)	–
MoCA	18.63 ± 4.39	26.64 ± 0.942	27.02 ± 1.17
**Language**			
Boston Naming Test**	20.02 ± 4.00	23.60 ± 3.32	25.47 ± 2.58
Verbal fluency test*	12.77 ± 3.19	14.84 ± 2.22	16.34 ± 2.45
**Attention and working memory**			
Trail Making Test A*	77.98 ± 30.28	72.64 ± 41.77	50.70 ± 20.33
Stroop color-word test**	69.19 ± 7.66	82.56 ± 5.45	84.53 ± 6.26
**Visuospatial function**			
Clock drawing task*	3.06 ± 0.98	3.82 ± 0.48	3.70 ± 0.689
Benton’s judgement of line* orientation	18.56 ± 5.04	22.68 ± 3.63	24.60 ± 4.38
**Memory**			
Rey–Osterrieth complex figure*	52.15 ± 16.66	74.44 ± 6.01	71.46 ± 12.11
Chinese auditory learning test*	8.75 ± 2.59	10.80 ± 2.32	10.79 ± 2.73
**Executive function**			
Numeric symbol conversion test*	21.48 ± 12.71	30.10 ± 5.73	31.28 ± 8.90
Tower of London test*	13.16 ± 5.41	17.30 ± 3.80	18.20 ± 3.55

*Compared to the HC group, the PDCI group showed a decrease of one standard deviation.

**Compared to the HC group, the PDCI group showed a decrease of two standard deviations. UPDRS, unified Parkinson’s disease rating scale; HY, modified Hoehn and Yahr clinical staging; MoCA, Montreal cognitive assessment.

### 2.1 Equipment

All participants were scanned using a 3.0T high-performance superconducting MRI system (uMR 770, Shanghai United Imaging Healthcare Co., Ltd.) equipped with a 24-channel head coil. Soft cushions were used to stabilize the participants’ heads to minimize motion artifacts. During image acquisition, participants were instructed to keep their eyes closed and remain awake. PD patients were scanned during the “on” phase of medication to minimize the effects of head motion on image quality.

### 2.2 Scan sequence parameters

All participants underwent 3D T1-weighted imaging (3D T1WI), T2-weighted imaging (T2WI), and T2 Fluid-Attenuated Inversion Recovery (T2 FLAIR) sequences to exclude other brain diseases, followed by QSM, and ASL scans. The 3D T1-weighted imaging utilized a magnetization-prepared rapid gradient echo sequence (3D T1WI GRE FSP) with the following parameters: field of view (FOV) 256 mm × 230 mm, matrix size 256 × 230, repetition time (TR) 6.94 ms, echo time (TE) 3.0 ms, flip angle 9°, slice thickness 1 mm, acquisition time 3 min 41 s. The ASL scan used a pseudo-continuous arterial spin labeling sequence (pCASL) with the following parameters: FOV 224 mm × 240 mm, matrix size 150 × 160, TR 5 s, TE 14.08 ms, flip angle 150°, labeling time 1,500 ms, post-labeling delay 2,001 ms, slice thickness 3 mm, acquisition time 4 min 36 s. The QSM scan used a gradient echo sequence (GRE) with the following parameters: voxel size 1 mm^3^ × 1 mm^3^ × 1 mm^3^, TR 28.3 s, TE 3.2 s, number of echoes 7, flip angle 15°, acquisition time 10 min.

### 2.3 Image preprocessing

ASL preprocessing: Whole-brain cerebral blood flow (CBF) maps for all participants were generated using the uNity 2.0 workstation. CBF values were calculated according to the recommended standards of the International Society for Magnetic Resonance in Medicine (ISMRM), with regional CBF expressed in units of mL/min/100 g (Alsop et al., 2015). For group-level analyses, all CBF images underwent spatial preprocessing using the SPM12 toolbox (MATLAB 2021b, MathWorks, Natick, MA, USA). First, the three-dimensional T1-weighted images (3D T1WI) and ASL images were manually reoriented to a common origin to ensure accurate spatial alignment. Subsequently, an affine transformation was applied to register each subject’s CBF map to the corresponding reoriented 3D T1 anatomical image (Fang et al., 2019). The 3D T1WI images were then segmented into gray matter, white matter, and cerebrospinal fluid (CSF) compartments. To minimize the influence of spatial deformation on signal intensity, modulation was applied during the segmentation process. Finally, the segmented 3D T1WI images and the co-registered CBF maps were normalized to the Montreal Neurological Institute (MNI) standard space (Despotović et al., 2015). The normalized CBF maps were subsequently smoothed with an isotropic Gaussian kernel of 8 mm full width at half maximum (FWHM) to improve the signal-to-noise ratio.

QSM preprocessing: QSM images were processed using the uNity 2.0 workstation, including region-of-interest extraction, phase unwrapping, background field removal, and magnetic dipole inversion (Fang et al., 2019; Wang and Liu, 2015). The QSM images and corresponding 3D T1WI images were manually reoriented to the same origin, followed by affine registration of each subject’s QSM image to the reoriented 3D T1 anatomical image. Spatial normalization to the standard template was then performed using the SPM12 toolbox. The normalized images were further processed using the data processing and analysis for brain imaging (DPABI) toolbox to enhance data consistency. Spatial smoothing was applied using an 8 mm FWHM Gaussian kernel, and gray matter masks were used to exclude white matter and CSF regions from subsequent analyses.

### 2.4 Statistical analysis

Participants were randomly divided into training and testing datasets at a ratio of 8:2. To prevent data leakage, the testing dataset was strictly excluded from the identification of brain regions exhibiting significant group differences. Within the training dataset, preprocessed QSM and ASL images were analyzed using the SPM12 toolbox, with voxel-wise multiple comparison correction performed using the family-wise error (FWE) method (cluster size > 500 voxels, *p* < 0.05). Significant brain regions were reported based on the Anatomical Automatic Labeling (AAL) atlas, and quantitative values were extracted from regions showing statistically significant differences among groups. Statistical analyses were conducted using SPSS version 27.0 (IBM Corp., Armonk, NY, USA). Age, sex, and years of education were included as covariates in regression analyses. Data normality was assessed using the Kolmogorov–Smirnov (K–S) test. For normally distributed data with homogeneity of variance, one-way analysis of variance (ANOVA) was used for group comparisons, followed by Bonferroni *post-hoc* tests for pairwise comparisons. When the assumption of equal variance was violated, the Tamhane *post-hoc* test was applied. Pearson correlation analysis was performed for normally distributed continuous variables, whereas Spearman correlation analysis was used for non-normally distributed or ordinal variables, with statistical significance defined as *p* < 0.05. Finally, QSM and CBF values extracted from brain regions exhibiting significant intergroup differences were used as input features for subsequent machine learning analyses.

### 2.5 Machine learning model construction

In this study, multiple classical machine learning classifiers were employed for classification analysis, including random forest (RF), K-nearest neighbors (KNN), XGB, decision tree (DT), Naive Bayes (NB), SVM, and logistic regression (LR). The detailed workflow is described as follows. First, feature matrices and corresponding labels were loaded, and the training and testing datasets were independently normalized using the MaxAbsScaler to minimize the influence of feature scale and distribution differences on model training (Cao et al., 2016). To address class imbalance, a class-weighting strategy was applied to all classifiers except the Naive Bayes model to balance the contribution of each class during training; for the Naive Bayes classifier, class imbalance was handled by adjusting the decision threshold. Subsequently, hyperparameter optimization was performed using GridSearchCV combined with 5-fold cross-validation, repeated over 100 iterations. During each grid search, model performance was evaluated via cross-validation, and the optimal hyperparameter configuration was selected based on the best validation performance (Kaliaev et al., 2025). To further assess the statistical significance of model performance, permutation testing was conducted. In addition, feature importance was quantified using the permutation_importance method, which estimates the relative contribution of each feature to model performance by measuring performance degradation after random feature shuffling (Ya et al., 2022). Finally, the optimal hyperparameter configuration identified from the training set was used to construct the final model, which was then evaluated on the independent test set. Evaluation metrics included test accuracy, area under the receiver operating characteristic curve (AUC), F1 score, sensitivity, specificity, and corresponding *p*-values. To further validate the robustness and statistical significance of the test-set performance, permutation testing was again applied, with 1,000 iterations conducted for each model. In each iteration, the *p*-value was calculated by comparing the true classification accuracy with the distribution of accuracies obtained from permuted labels, and the mean *p*-value across the 1,000 iterations was reported as the final measure of statistical significance.

## 3 Results

ANOVA across the three groups revealed significant differences in CBF and magnetic susceptibility between the HC group and both PDCI and PDNCI groups, whereas no significant differences were observed between the PDCI and PDNCI groups.

### 3.1 PDCI group vs. HC group

As summarized in Table 1, compared with the HC group, patients in the PDCI group demonstrated declines of at least one standard deviation across multiple cognitive subdomains, including language, attention and working memory, visuospatial function, and executive function. The most pronounced impairments were observed in the language domain, assessed using the Boston Naming Test, and in the attention and working memory domain, evaluated by the Stroop Color–Word Test, both of which showed declines exceeding two standard deviations relative to healthy controls (Mack and Marsh, 2017; Weil et al., 2018).

**FIGURE 1:**
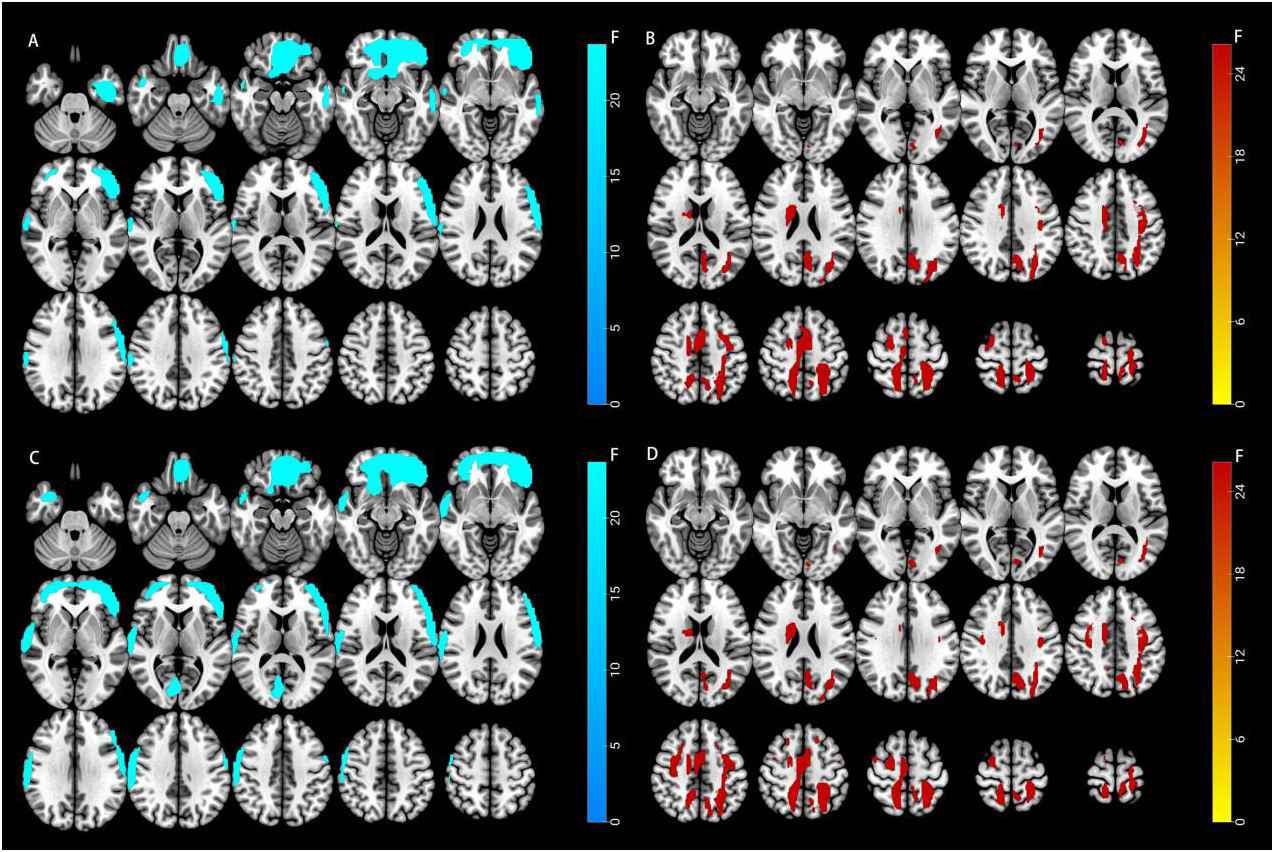
Distribution of brain regions with changes in cerebral blood flow (CBF) and magnetic susceptibility in the PDCI and PDNCI groups compared to HC. **(A)** Brain regions with reduced CBF in the PDCI group; **(B)** Brain regions with increased magnetic susceptibility in the PDCI group; **(C)** Brain regions with reduced CBF in the PDNCI group; **(D)** Brain regions with increased magnetic susceptibility in the PDNCI group (*p* < 0.05).

When analyzing the CBF values between the two groups, we found significant differences in brain perfusion between PDCI and HC. Compared to the HC group, the PDCI group showed reduced CBF values in brain regions such as Frontal _Sup _Orb _L, Frontal _Mid _Orb _L, Frontal _Inf _Tri _L, Frontal _Inf _Orb _L, Temporal _Mid _L, and Temporal _Inf _L (Figure 1A). In contrast, increased magnetic susceptibility was observed in regions such as Occipital _Mid _L, Postcentral _L, Parietal _Sup _L, and Precuneus _L (Figure 1B).

**FIGURE 2:**
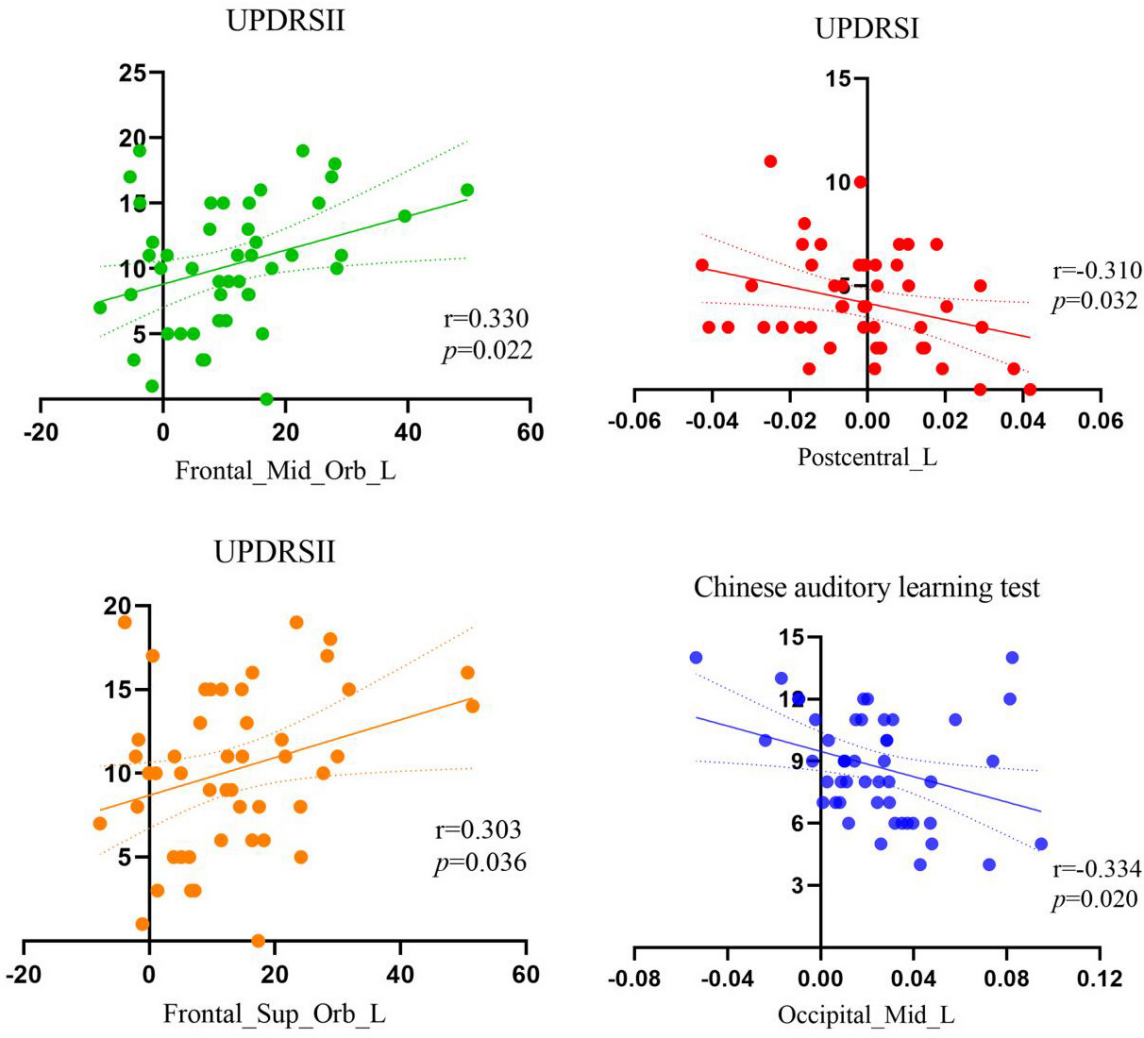
Pearson correlation analysis results in the PDCI group (*p* < 0.05). UPDRS, unified Parkinson’s disease rating scale.

As shown in Figure 2, Pearson correlation analysis was performed between the PDCI group and clinical scales that followed a normal distribution. The results revealed that the UPDRS I score was positively correlated with the CBF values of Frontal_Mid_Orb_L, while the UPDRS II score was positively correlated with the CBF values of Frontal_Mid_Orb_L and negatively correlated with the magnetic susceptibility of Postcentral_L. Additionally, the Chinese auditory learning test score was negatively correlated with the magnetic susceptibility of Occipital_Mid_L.

**FIGURE 3:**
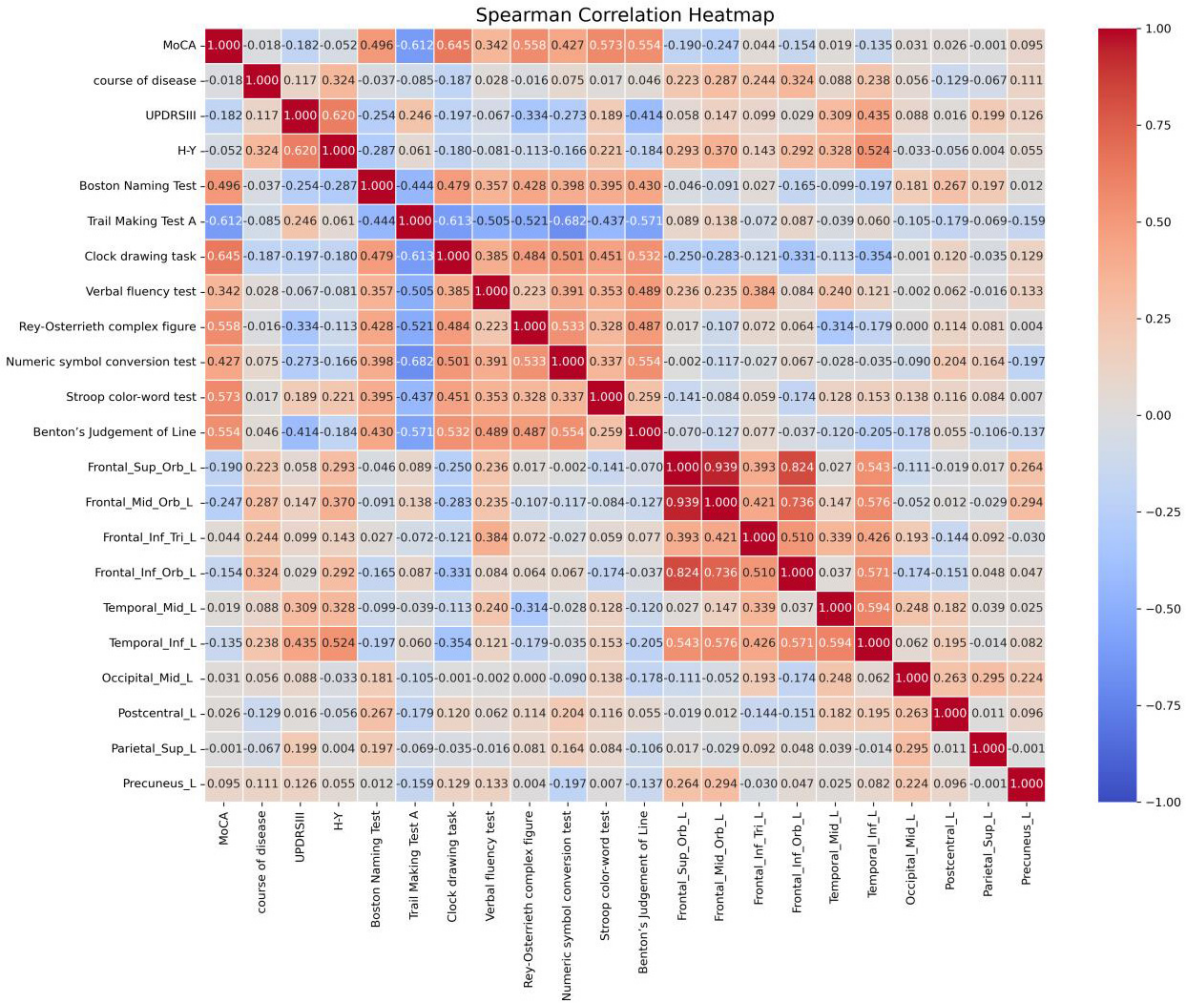
Spearman correlation analysis results in the PDCI group (*p* < 0.05). UPDRS, unified Parkinson’s disease rating scale; HY, modified Hoehn and Yahr clinical staging; MoCA, Montreal cognitive assessment.

In contrast, Spearman correlation analysis was performed for clinical scales that did not follow a normal distribution, as shown in Figure 3. This analysis revealed that disease duration was positively correlated with the CBF values of Frontal_Mid_Orb_L and Frontal_Inf_Orb_L. The UPDRS III score was positively correlated with the scores of Temporal_Mid_L and Temporal_Inf_L. The H-Y score showed a positive correlation with the CBF values of Frontal_Sup_Orb_L, Frontal_Mid_Orb_L, Frontal_Inf_Orb_L, and Temporal_Inf_L. Finally, the range fluency score was positively correlated with the CBF values of Frontal_Inf_Tri_L.

**FIGURE 4:**
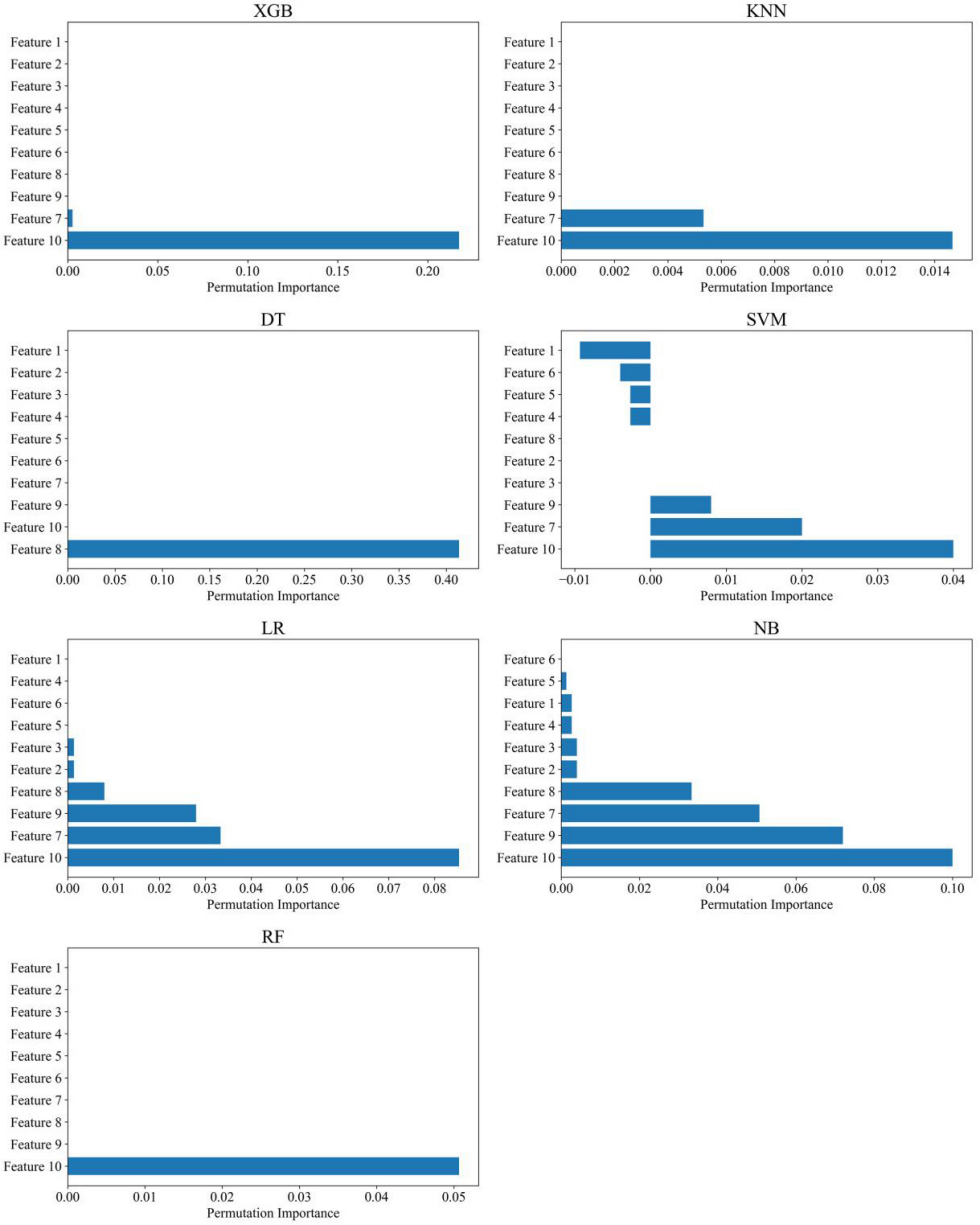
Feature importance of various machine learning models in the PDCI group. RF, random forest classifier; KNN, K-nearest neighbors classifier; XGB, Extreme Gradient Boosting Classifier; DT, decision tree classifier; NB, Naive Bayes classifier; SVM, support vector machine classifier; LR, logistic regression classifier. Feature 1 = Frontal_Sup_Orb_L (Left superior orbital frontal cortex); Feature 2 = Frontal_Mid_Orb_L (Left middle orbital frontal cortex); Feature 3 = Frontal_Inf_Tri_L (Left inferior triangular frontal cortex); Feature 4 = Frontal_Inf_Orb_L (Left inferior orbital frontal cortex); Feature 5 = Temporal_Mid_L (Left middle temporal cortex); Feature 6 = Temporal_Inf_L (Left inferior temporal cortex); Feature 7 = Occipital_Mid_L (Left middle occipital cortex); Feature 8 = Postcentral_L (Left postcentral gyrus); Feature 9 = Parietal_Sup_L (Left superior parietal cortex); Feature 10 = Precuneus_L (Left precuneus cortex). Features 1 to 6 correspond to brain regions with reduced cerebral blood flow in the PDCI group, while Features 7 to 10 correspond to brain regions with increased magnetic susceptibility in the PDCI group.

The disease diagnostic models built using machine learning techniques demonstrated excellent performance in distinguishing PDCI from HC. Among the models, RF, KNN, and LR performed the best, with all metrics reaching 1.000 (Table 2). Furthermore, we found that increased magnetic susceptibility in the Precuneus _L and Postcentral _L regions was the most important feature for distinguishing PDCI (Figure 4).

**TABLE 2:** Machine learning results based on PDCI multimodal features from ASL and QSM.

Model	Test Acc	AUC	F1 score	Sensitivity	Specificity	*p*-value
RF	1.000	1.000	1.000	1.000	1.000	0.001
KNN	1.000	1.000	1.000	1.000	1.000	0.001
XGB	0.950	1.000	0.947	0.900	1.000	0.001
DT	0.900	0.980	0.889	0.800	1.000	0.002
NB	0.950	1.000	0.947	0.900	1.000	0.001
SVM	0.950	1.000	0.947	0.900	1.000	0.001
LR	1.000	1.000	1.000	1.000	1.000	0.001

RF, random forest; KNN, K-nearest neighbors; XGB, Extreme Gradient Boosting; DT, decision tree; NB, Naive Bayes; SVM, support vector machine; LR, logistic regression. Test Acc, test accuracy; AUC, area under the receiver operating characteristic curve. The *p*-value is calculated as the average across 1,000 iterations of permutation tests.

### 3.2 PDNCI group vs. HC group

**FIGURE 5:**
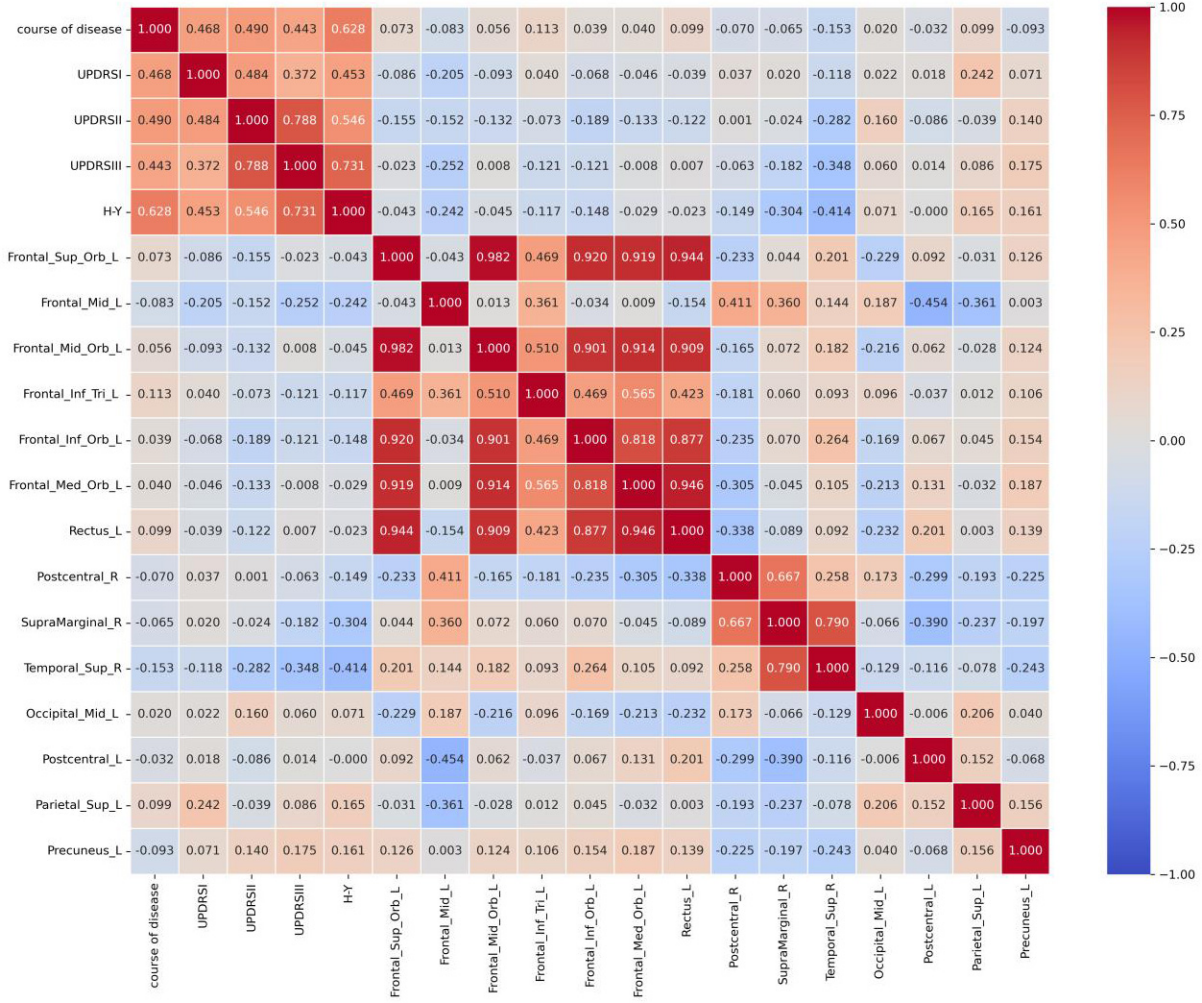
Spearman correlation analysis results in the PDNCI group (*p* < 0.05). UPDRS, unified Parkinson’s disease rating scale; HY, modified Hoehn and Yahr clinical staging.

Analysis of CBF values revealed significant differences in cerebral perfusion between the PDNCI and HC groups. Compared with the HC group, the PDNCI group exhibited significantly reduced CBF in Frontal_Sup_Orb_L, Frontal_Mid_L, Frontal_Mid_Orb_L, Frontal_Inf_Tri_L, Frontal_Inf_Orb_L, Frontal_Med_Orb_L, Rectus_L, Postcentral_R, SupraMarginal_R, and Temporal_Sup_R (Figure 1C). In contrast, significantly increased magnetic susceptibility was observed in Occipital_Mid_L, Postcentral_L, Parietal_Sup_L, and Precuneus_L (Figure 1D). Correlation analyses between imaging features and clinical scales in the PDNCI group demonstrated that UPDRS II scores were positively correlated with CBF values in Temporal_Sup_R, while UPDRS III scores also showed a positive correlation with CBF values in Temporal_Sup_R. In addition, H–Y stage was positively correlated with CBF values in SupraMarginal_R and Temporal_Sup_R (Figure 5).

**FIGURE 6:**
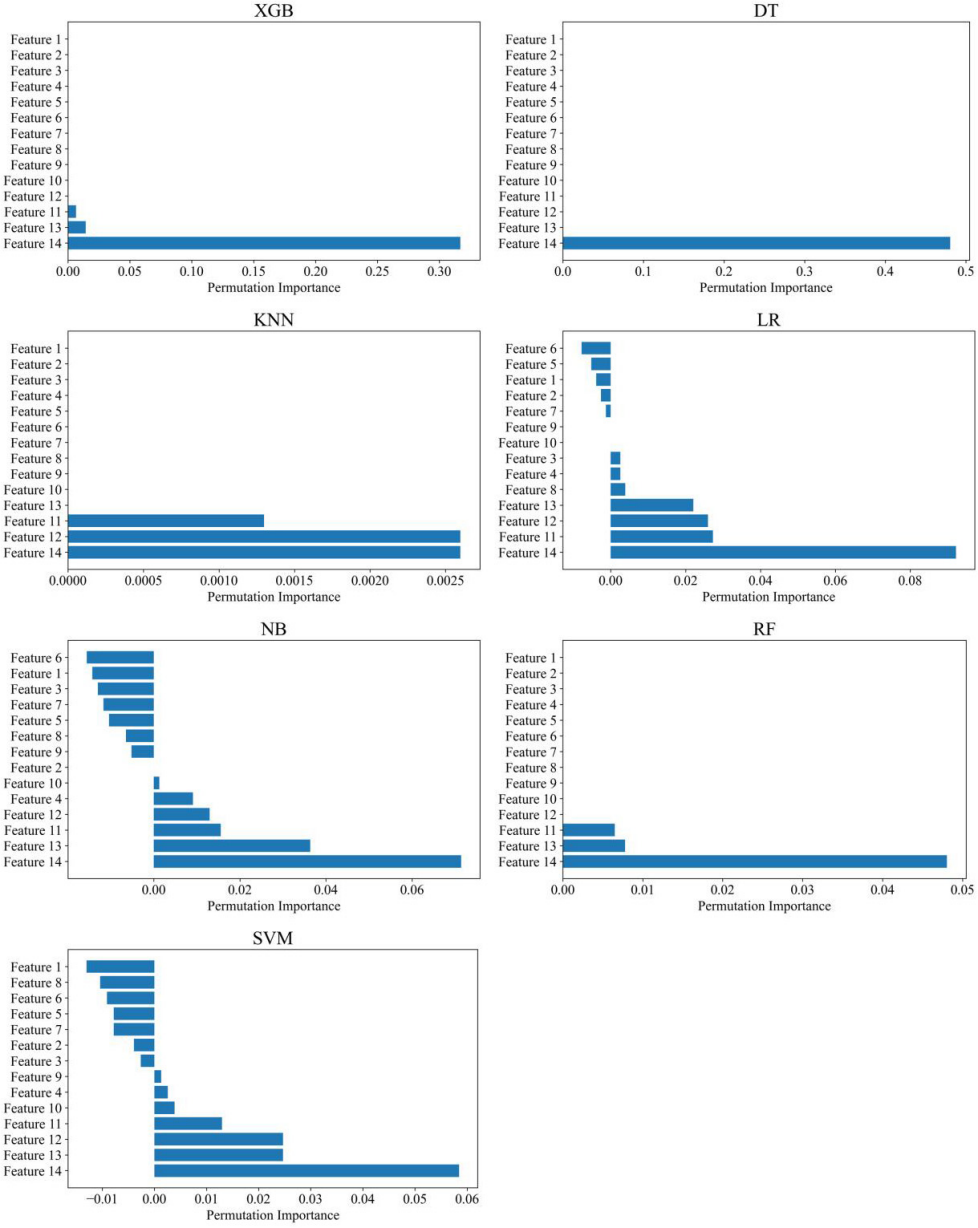
Feature importance of various machine learning models in the PDNCI group. RF, random forest classifier; KNN, K-nearest neighbors classifier; XGB, Extreme Gradient Boosting Classifier; DT, decision tree classifier; NB, Naive Bayes classifier; SVM, support vector machine classifier; LR, logistic regression classifier. Feature 1 = Left superior orbital frontal cortex (Frontal_Sup_Orb_L), Feature 2 = Left middle frontal cortex (Frontal_Mid_L), Feature 3 = Left middle orbital frontal cortex (Frontal_Mid_Orb_L), Feature 4 = Left inferior triangular frontal cortex (Frontal_Inf_Tri_L), Feature 5 = Left inferior orbital frontal cortex (Frontal_Inf_Orb_L), Feature 6 = Left medial orbital frontal cortex (Frontal_Med_Orb_L), Feature 7 = Left rectus (Rectus_L), Feature 8 = Right postcentral gyrus (Postcentral_R), Feature 9 = Right supramarginal gyrus (SupraMarginal_R), Feature 10 = Right superior temporal cortex (Temporal_Sup_R), Feature 11 = Left middle occipital cortex (Occipital_Mid_L), Feature 12 = Left postcentral gyrus (Postcentral_L), Feature 13 = Left superior parietal cortex (Parietal_Sup_L), Feature 14 = Left precuneus cortex (Precuneus_L). Features 1 to 10 correspond to brain regions with reduced cerebral blood flow in the PDNCI group, while Features 11 to 14 correspond to brain regions with increased magnetic susceptibility in the PDNCI group.

The machine learning–based diagnostic models also demonstrated excellent performance in discriminating PDNCI from HC. Among all classifiers, RF, KNN, and XGB achieved the best performance, with all evaluation metrics reaching 1.000 (Table 3). Feature importance analysis further indicated that increased magnetic susceptibility in Precuneus_L was the most influential feature for distinguishing PDNCI (Figure 6).

**TABLE 3:** Machine learning results based on PDNCI multimodal features from ASL and QSM.

Model	Test Acc	AUC	F1 score	Sensitivity	Specificity	*p*-value
RF	1.000	1.000	1.000	1.000	1.000	0.001
KNN	1.000	1.000	1.000	1.000	1.000	0.001
XGB	1.000	1.000	1.000	1.000	1.000	0.001
DT	0.950	0.940	0.947	0.900	1.000	0.001
NB	0.950	1.000	0.947	0.900	1.000	0.001
SVM	0.950	0.990	0.947	0.900	1.000	0.001
LR	0.950	1.000	0.947	0.900	1.000	0.001

RF, random forest; KNN, K-nearest neighbors; XGB, Extreme Gradient Boosting; DT, decision tree; NB, Naive Bayes; SVM, support vector machine; LR, logistic regression; Test Acc, test accuracy; AUC, area under the receiver operating characteristic curve. The *p*-value is calculated as the average across 1,000 iterations of permutation tests.

## 4 Discussion

The results of this study indicate that patients with PDCI exhibit significant cognitive decline across multiple domains, including language, attention and working memory, visuospatial ability, executive function, and memory. Among these domains, language impairment was particularly prominent, as reflected by marked reductions in performance on the Boston Naming Test and the Verbal Fluency Test, suggesting deficits in naming ability and semantic fluency. In the present study, Verbal Fluency Test performance in PD patients was positively correlated with CBF values in Frontal_Inf_Tri_L, a region closely associated with executive control and language processing. This finding is consistent with the established role of the prefrontal cortex in language production and executive function, particularly the involvement of the left inferior frontal region in speech fluency and verbal generation (Levy et al., 2025). The prefrontal cortex plays a critical role in language generation and monitoring, and reduced perfusion in this region may directly impair semantic retrieval and speech planning processes (Heo et al., 2023).

In the domains of attention and working memory, the PDCI group demonstrated significant impairments on the Trail Making Test A and the Stroop Color–Word Test, reflecting deficits in cognitive flexibility, task switching, and interference control. These cognitive functions are primarily mediated by the frontoparietal network, and ASL findings in this study revealed significantly reduced perfusion within these regions. Such hypoperfusion may disrupt the synchronization and integration of neuronal signaling, thereby impairing attentional control and working memory processes (Yang et al., 2023). Moreover, increased iron deposition in frontal and parietal regions has been reported to exacerbate deficits in attention and response inhibition by promoting oxidative stress and damaging dopaminergic pathways, ultimately accelerating neurodegenerative processes (Thomas et al., 2020).

Visuospatial dysfunction was also evident in patients with PDCI. Performance on the Clock Drawing Test and Benton’s Judgment of Line Orientation Test revealed marked impairments in spatial construction, perceptual processing, and spatial relationship judgment. These deficits were closely associated with reduced perfusion in the parietal and occipital lobes, where ASL demonstrated significantly decreased CBF, while QSM revealed increased magnetic susceptibility. These findings suggest that abnormal iron accumulation in these regions may further exacerbate metabolic dysfunction and impair neuronal signal transmission. Iron deposition in the parietal–occipital cortex has been proposed to disrupt the balance between excitatory and inhibitory neural activity, thereby compromising the integration and construction of complex visuospatial information (Mohammadi and Ghaderi, 2024).

Executive dysfunction is one of the most clinically prominent features of PDCI. In this study, the PDCI group exhibited significant impairments on the Numeric Symbol Conversion Test and the Tower of London Test, reflecting deficits in planning, reasoning, and problem-solving abilities. These impairments were primarily associated with decreased CBF and increased magnetic susceptibility in prefrontal regions, including Frontal_Mid_L and Frontal_Inf_Orb_L, suggesting a reduction in the metabolic capacity of the cognitive control network. These findings are consistent with previous evidence indicating that executive dysfunction arises from disruptions in the prefrontal–striatal–thalamic circuitry, with abnormal iron deposition further exacerbating this pathological process (Mohammadi and Ghaderi, 2024).

With respect to memory function, performance on the Rey–Osterrieth Complex Figure Test and the Chinese Auditory Learning Test (CALT) revealed significant memory deficits in the PDCI group. Notably, CALT scores in PD patients with cognitive impairment were negatively correlated with magnetic susceptibility in Occipital_Mid_L, suggesting that functional abnormalities in this region may be closely associated with impairments in auditory learning and memory. Although the occipital lobe is traditionally regarded as a primary visual processing region, an increasing body of evidence has demonstrated its involvement in multisensory integration and higher-order cognitive processes (Wei et al., 2024). In particular, the left occipital lobe has been implicated in language and memory-related tasks (Ma et al., 2024), and magnetic susceptibility, as an indirect marker of microstructural alterations, may reflect functional damage to the neural networks supporting these cognitive functions (Thomas et al., 2020).

In addition, several clinical scales used in this study, including UPDRS II, UPDRS III, and H–Y stage, exhibited correlations with CBF; however, these scales primarily reflect the severity of motor symptoms and disease stage rather than direct measures of cognitive impairment. The correlation between disease duration and CBF in Frontal_Mid_Orb_L, as well as the positive association between UPDRS II scores and CBF in this region, may not directly indicate cognitive deterioration but could represent compensatory perfusion changes occurring at specific stages of disease progression, potentially delaying functional decline. Similarly, the positive correlations between UPDRS III scores and CBF in Temporal_Mid_L and Temporal_Inf_L, and between H–Y stage and CBF across multiple frontal and temporal regions, likely reflect the broader impact of motor system pathology on cerebral blood flow regulation and metabolic demand (Liu et al., 2022). These findings suggest that progression of motor dysfunction may be accompanied by adaptive changes in regional perfusion. In the PDCI group, UPDRS II scores were negatively correlated with magnetic susceptibility in the postcentral gyrus, indicating that microstructural alterations in this region may be closely related to declining motor function. Furthermore, sensory dysfunction may also influence cognitive performance, particularly as motor impairments worsen, thereby contributing to reduced independence in daily activities (Aarsland et al., 2021). In PDNCI patients, UPDRS II, UPDRS III, and H–Y stage were positively correlated with CBF in Temporal_Sup_R and SupraMarginal_R, suggesting that even at relatively early stages of cognitive involvement, cognitive-related brain regions may participate in functional compensation within the motor network.

In the present study, no significant differences were observed between the PDCI and PDNCI groups in ASL-derived cerebral blood flow (CBF) or QSM-based magnetic susceptibility measures. Although this finding may initially appear inconsistent with the primary aim of distinguishing cognitive subtypes of Parkinson’s disease using multimodal MRI features, it is biologically plausible when interpreted in the context of existing literature and the pathophysiology of PD-related cognitive impairment. Cognitive impairment in PD is widely regarded as a heterogeneous and continuously evolving process rather than a strictly dichotomous condition, and the diagnosis of PDCI or PD-MCI relies largely on neuropsychological thresholds (e.g., MoCA < 26 or performance 1–2 standard deviations below normative values), which vary across studies and do not necessarily correspond to discrete neurobiological stages (Litvan et al., 2012). Moreover, different cognitive phenotypes—such as executive, language, visuospatial, or memory impairment—are associated with distinct neural network dysfunctions, and their imaging effects may be averaged out in group-level analyses, thereby reducing the detectability of differences between PDCI and PDNCI. From the perspective of cerebral perfusion, previous ASL studies consistently demonstrate widespread cortical hypoperfusion in PD patients compared with healthy controls, particularly in frontal, temporal, and parietal regions, with strong associations with global cognitive decline (Melzer et al., 2011); however, perfusion differences between cognitively normal and cognitively impaired PD patients remain inconsistent, especially in the early to middle stages of the disease, where such differences are subtle, spatially heterogeneous, and sensitive to factors such as sample size, disease duration, medication status, and analytical strategies, including stringent FWE correction. The relatively modest sample size of the present study further limits statistical power to detect small-to-moderate effect sizes between closely related phenotypes, and although voxel-wise FWE correction effectively controls type I error, it may reduce sensitivity to biologically meaningful but subtle effects, increasing the risk of false-negative findings. Similarly, QSM studies have demonstrated increased magnetic susceptibility in multiple cortical and subcortical regions in PD and its association with cognitive decline (Shibata et al., 2022), yet evidence regarding its ability to distinguish PD-MCI from cognitively normal PD patients remains mixed, with some findings suggesting that susceptibility changes primarily reflect global PD-related neurodegenerative processes rather than stage-specific cognitive markers (Melzer et al., 2011; Zhao et al., 2022). In addition, cognitive impairment in PD arises from multiple interacting pathological mechanisms, including α-synuclein pathology, cholinergic dysfunction, coexisting Alzheimer’s disease–related pathology, and cerebrovascular contributions (Kehagia et al., 2013), implying that single-modality imaging measures such as ASL or QSM alone may be insufficient to fully capture the neurobiological complexity underlying cognitive status in a cross-sectional setting. Taken together, the presence of significant ASL and QSM differences between healthy controls and PD patients, but not between PDCI and PDNCI, suggests that these imaging metrics in the present cohort and analytical framework may be more sensitive to global PD-related alterations in cerebral perfusion and iron metabolism rather than serving as highly specific biomarkers for differentiating cognitive subtypes defined by neuropsychological thresholds; future studies incorporating larger sample sizes, longitudinal follow-up, and more advanced network-based, radiomic, and multimodal integration approaches may further improve the detection and interpretation of the neurobiological substrates underlying cognitive heterogeneity in Parkinson’s disease.

In this study, several machine learning models were constructed based on ASL- and QSM-derived imaging features to discriminate among PDCI, PDNCI, and HC. Overall, all models achieved high classification performance, with KNN, RF, and XGB showing the best discriminative ability, as reflected by AUC, F1-score, sensitivity, and specificity values all reaching 1.000. Feature importance analysis further revealed that increased magnetic susceptibility in Precuneus_L and Postcentral_L was the most influential feature for distinguishing PDCI, highlighting the potential relevance of these regions in PD-related cognitive impairment.

Nevertheless, the excellent performance of the models should be interpreted with caution. Given the relatively limited sample size, there remains a potential risk of overfitting, whereby the models may capture sample-specific patterns that do not generalize well to larger or independent datasets. Overfitting is particularly likely in scenarios involving high-dimensional feature spaces and limited sample sizes, where models may inadvertently learn noise or individual variability rather than robust disease-related patterns (Rezapoor et al., 2025). Although strategies such as 5-fold cross-validation and permutation testing were employed to mitigate the influence of random variation, an overestimation of model performance cannot be entirely excluded under small-sample conditions. Therefore, the present findings should be regarded as exploratory, demonstrating the potential diagnostic value of ASL and QSM imaging features for identifying PDCI and PDNCI, rather than as definitive or clinically deployable diagnostic models.

From a biological perspective, the excellent performance of the machine learning models also reflects meaningful differences in cerebral perfusion and magnetic susceptibility characteristics between PDCI and PDNCI patients. Feature importance analyses consistently identified Precuneus_L and Postcentral_L as the most influential regions contributing to classification performance. Increased magnetic susceptibility in these regions has been closely associated with cognitive dysfunction, indicating that the model decision-making process is supported by clear physiological plausibility. Thus, machine learning not only enables high-precision classification at the pattern-recognition level but also provides quantitative insights into the pathophysiological mechanisms underlying PDCI.

Moreover, feature importance analyses further revealed distinct regional patterns across different clinical groups. In the PDCI group, increased magnetic susceptibility in Precuneus_L and Postcentral_L emerged as the most representative discriminative features, suggesting that abnormal iron metabolism in parietal regions—areas critically involved in visuospatial integration and spatial representation—may substantially contribute to higher-order cognitive impairment at more advanced stages (Thomas et al., 2020). In contrast, classification models for the PDNCI group predominantly relied on increased magnetic susceptibility in Precuneus_L, implying that even at relatively early stages of cognitive involvement, subtle metabolic imbalance and functional decline in this region can be detected by machine learning approaches, potentially serving as an early imaging marker of cognitive vulnerability (Shibata et al., 2022). Together, these findings provide layered physiological evidence for progressive brain functional alterations across different cognitive stages of PD and highlight the sensitivity of QSM-derived metrics in capturing early neurodegenerative changes.

Overall, the machine learning results suggest that multimodal imaging features integrating ASL and QSM hold substantial potential for the early identification of PDCI and PDNCI. Future studies should incorporate larger sample sizes, independent validation cohorts, and longitudinal follow-up to further assess the robustness and generalizability of these models. In addition, advanced feature selection and interpretation techniques, such as LASSO regression and SHAP value analysis, may enhance model interpretability and facilitate a more precise understanding of how alterations in cerebral perfusion and iron deposition contribute to cognitive dysfunction in PD. With support from large-scale, multicenter investigations, this multimodal imaging and machine learning framework may ultimately evolve into an auxiliary tool for diagnosis and disease stratification in PD with cognitive impairment, offering a promising pathway toward precision medicine.

## 5 Conclusion

ASL- and QSM-derived imaging features demonstrate substantial potential as non-invasive biomarkers for the early identification of PDCI. Patients with PDCI exhibit widespread impairments across multiple cognitive domains, particularly in language, attention, and working memory. Machine learning models integrating multimodal imaging features show strong capability in distinguishing different cognitive states in Parkinson’s disease, highlighting their potential value for early diagnosis and individualized disease assessment. Nevertheless, future studies with larger sample sizes, independent validation cohorts, and longitudinal designs are warranted to further improve the robustness, stability, and generalizability of these models before clinical application.

## Data Availability

The raw data supporting the conclusions of this article will be made available by the authors, without undue reservation.
